# “Expression of genes encoding protein disulfide isomerase (PDI) in cultivars and lines of common wheat with different baking quality of flour”

**DOI:** 10.1186/s12870-018-1522-z

**Published:** 2018-11-22

**Authors:** Katarzyna Demska, Ewa Filip, Lidia Skuza

**Affiliations:** 1 Department of Cell Biology, Faculty of Biology, The Institute for Research on Biodiversity, University of Szczecin, Wąska 13, 71-415 Szczecin, Poland; 2 The Centre for Molecular Biology and Biotechnology, Wąska 13, 71-415 Szczecin, Poland

**Keywords:** Molecular biology, Gene expression, Wheat, Gluten

## Abstract

**Background:**

The subject of this research was to investigate the level of expression of genes encoding protein disulfide isomerase (PDI) in cultivars and lines of wheat with different baking value of flour. PDI plays a key role in the formation of disulfide bonds in newly formed proteins. Each of cultivars and lines had a specific set of high molecular weight glutenin subunits (HMW-GS). Based on the presence of individual subunits, the potential baking value is predicted. Sometimes this value is not confirmed during technological analysis. Since there are cases where flour has a better or worse value than expected on the basis of the genotype, the expression of PDI genes was considered as a potential cause for discrepancies mentioned.

**Results:**

Analysis focused on three stages of grain development. The expression level of PDI genes was compared between wheat cultivars and lines with different genotype-phenotype combinations, which means diversified sets of HMW-GS combined with diversified qualitative classification. The highest expression level of PDI was noticed at early stage of grain development, which is consistent with the function of PDI. The expression level was evaluated by the real-time PCR technique.

**Conclusion:**

Results obtained in this work did not allow for a clear statement of decisive significance of PDI in the context of shaping the final baking value. The results of this work contribute to an ever more in-depth understanding of the mechanisms governing baking value, and thus to the progress of the selection of new varieties with more beneficial properties.

## Background

Baking value assessment of wheat flour is important in the context of the utility importance of this cereal. There is a set of technological indicators, the use of which allows to determine flour quality and value, enabling the assessment and determination of the intended use of tested wheat genotypes, and thereby selecting components for high-quality cultivars [1]. Flour of various qualities are used, depending on their purpose, hence the information on their baking suitability is very important.

In Poland, the utility classification of wheat cultivars is determined by COBORU (Research Centre for Cultivar Testing) based on research involving the determination of specific technological indicators of flour and grain. Five wheat quality groups have been created in relation to their purpose starting from the best: E – elite (exclusive), A – qualitative, B – bread, K – cake (cookies), and C – remained. Group E is formed by varieties with best quality. Group A contains varieties which have good baking quality. In group B there are varieties used for bread baking. In group K there are varieties used for preparing cakes or cookies. The rest of varieties including these used for fodder are gathered in group C. Assignment to one of above-mentioned specific groups is based on the so-called multi-feature method. There are several well-known features and rheological properties which are very important for the classification, i.a. sedimentation index, protein content, water absorption of flour, dough energy, bread volume. These traits result directly from physicochemical properties of gluten and have crucial role in creating baking value. For each of several parameters indicated by COBORU, nine ranges of values were established. The most favorable range of values obtained for a given indicator is referred to as class 9, while the least desirable is class 1. Each variety is classified in one of nine classes in terms of each parameter. Each of the quality groups (E, A, B, K and C) has got of the quality groups has minimum class requirements for each of the 9 parameters. It goes as follows: for example a given variety may be included in the best qualitative group E, if the value of the sedimentation index allows it to be classified at least in class 7 of nine. To include this variety in the quality group A, it is enough that in terms of this parameter it will be in class 5.

As mentioned above, cultivar classification to a given technological group is determined by flour quality, and this depends primarily on gluten proteins. Gluten consists of several fractions present in different proportions. These fractions are monomeric gliadins and polymeric glutenins. Each of them in turn includes several types, differing in their properties, such as molecular weight, electrophoretic mobility etc. Gliadins dissolve in 60–70% alcohol, while glutenins are insoluble in these solutions [2–4]. Glutenins were divided into high molecular weight glutenin subunits (HMW-GS) with a mass of approximately 70–90 kDa, and low molecular weight glutenin subunits (LMW-GS) with a mass of around 20–45 kDa [2, 5]. Type x and type y were distinguished in HMW that differ in electrophoretic migration rate.

HMW glutenins are considered the most important gluten fraction. They largely determine gluten quality, and thus also the rheological characteristics of dough formation, i.e., viscosity, elasticity, strength, expansibility, etc. [6]. The loci of the *Glu-1* genes, encoding the HMW subunits, are located on the long arms of the first group chromosomes: 1A, 1B and 1D (in wheat genomes A, B and D). Each locus contains two genes encoding the HMW type: x and y. An individual cultivar produces proteins that form gluten with a slightly different structure and properties, depending on the set of alleles and the presence of particular HMW subunits, which translates into different flour quality. The final baking value is also influenced by environmental factors affecting wheat in the successive phases of its growth, and thus also the grain formation and maturation processes. Nevertheless, the importance of genetic determinants of a given cultivar baking properties is widely emphasized [3, 7].

To determine the usefulness of the wheat genotype for baking purposes, Payne and Lawrence [8] provided basic classification of the alleles present in the above loci. They assigned a specific number of points (so-called quality points) to each subunit and created a ten-point quality scale. The sum of quality points for a given cultivar is supposed to reflect its baking value. There are characteristic subunits for each genome, considered as markers of baking value. The baking quality of a given cultivar can be estimated based on the presence of particular subunits in three genomes. Common wheat varieties and lines which give high-quality flour include in their D genome the set of alleles encoding HMW subunits Dx5 + Dy10, whereas the presence of the set Dx2 + Dy12 is a marker which determines in a certain sense worse baking quality [9]. In the B genome the set of subunits Bx7 + By9 is desirable, but the set Bx6 + By8 adversely affect the baking quality. In the A genome presence of the subunit Ax1 is advantageous [7]. The occurrence of particular subunits or glutenin protein blocks is often verified by SDS-PAGE separation or PCR. On the basis of the presence of particular subunits in the three genomes we might potentially predict the baking quality of grain derived from given variety. It has been demonstrated many times that the presence of ‘favorable’ or ‘unfavorable’ subunits correlates with the desired or unwanted values of technological parameters, i.e., gluten index, sedimentation test and others [7, 10, 11].

Payne and Lawrence quality scale was created in order to serve to predict and determine baking quality of individual wheat varieties and lines. The scale is based on scientific knowledge about markers of good or worse baking quality. As we mentioned above, there are well-known markers which means occurrence of some HMW glutenin subunits may help to predict to some extent baking quality of given wheat variety. For example the presence of alleles encoding HMW Dx5 + Dy10 glutenin subunits means 4 points on a 10-point scale. On the contrary, occurrence Dx2 + Dy12 as a marker of worse quality is connected with only 2 points. Favorable are also subunits Bx7 + By8 and Ax2* (3 points). With worse quality are connected subunits Bx6 + By8 or Ax Null (1 point). After adding points from individual genomes, we obtain the total score for the wheat variety. For example total score for Zyta is 7 (3 + 2 + 2) because of its genotype 2*/7 + 9/2 + 12 (Table 2).

**Fig. 1:**
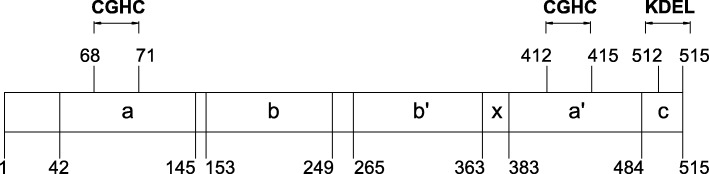
Structure of protein disulfide isomerase in wheat (based on [43])

Valuable glutenin HMW and LMW glutenin subunits are constantly sought for, and the content of gliadins, especially of the ω and γ type, is being investigated, because these proteins are the basis of gluten physicochemical properties. The presence of disulfide bonds in gluten proteins is also important for dough forming processes. In monomeric gliadins, 3–4 intrachain bonds are found in polypeptide chains, and in polymeric glutenins there are intra- as well as interchain bonds [2]. Protein disulfide isomerase (PDI) enables formation of such a polymeric gluten network. Its structure is mainly dependent on the thioredoxin (TRX) protein superfamily that PDI belongs to, where the characteristic spatial system – thioredoxin fold – is a specific feature, which is a fragment of thioredoxin tertiary structure [12]. Several domains can be distinguished in the PDI structure (Fig. 1). An active center containing the CGHC (Cys-Gly-His-Cys) motif is present within the a and a’ domains. Cysteine residues are involved in the rearrangement and formation of disulfide bonds in proteins. The most important structural feature of the b and b’ domains is the presence of a peptide-binding site, among which are new proteins waiting for folding, as well as those folded inappropriately [12].

PDI is the first identified catalyst for protein maturation and folding [13]. It is a complex process, and therefore, prone to errors, especially in case of longer chains. Sometimes in wrongly folded chains there is an exposure of regions that are normally hidden. This does not lead then to a native conformation. The presence of chaperone proteins, including PDI is a defensive mechanism [14].

The loci for sequences coding PDI are located in the fourth group of homeologous chromosomes, and the coding sequences are named: *PDI-4A*, *PDI-4B* and *PDI-4D* [15–17]. PDI sequences were examined for molecular purposes in hexaploid wheat and its evolutionary ancestors [17]. Coding sequences in various tissues, gene sequences and promoters in wheat and its ancestors were also examined [16, 18, 19]. Literature additionally provides the results of research on the qualitative characterization of cultivars both in molecular and technological terms [9, 20, 21]. However, no studies linking these results to PDI expression were performed to determine the possible indirect effect of PDI expression on baking value.

Usually information obtained from the genotype analysis is consistent with obtained rheological properties of dough and flour. Sometimes, unfortunately varieties give a flour which has a quality other (better or worse) than expected on the basis of genotype investigation. There are cases when the results of technological research show a different baking value of flour from grains of a given cultivar than expected based on molecular research of the genotype. This may result from abnormalities occurring during the maturation of proteins or adopting a wrong and/or unstable conformation. The total number of points in the qualitative Payne and Lawrence scale does not provide full information about the baking value. Nevertheless, a wide range of knowledge about connections of baking quality with specific HMW subunits gathered over the years still remains a strong determinant in this area. The genotype is often examined for the presence of good and inferior baking value markers, and sometimes such analyses are combined with technological research. In contrast, PDI data focus mainly on the level of expression of genes encoding it without reference to the baking value. Considering the role that disulfide isomerase plays in the maturation of gluten proteins, the aim of the study was to examine the expression of PDI in three wheat genomes in cultivars and lines with different flour baking values.

## Results

### Semi-quantitative analysis of PDI expression

One specific fragment of the expected size was obtained as a result of fragment amplification of the genes coding for PDI and CDC48 for all samples (cultivars and lines) within each collection. There were visible differences in the intensity of the products for individual wheat genotypes at various stages of caryopsis development (Table 1).

**Table 1:**
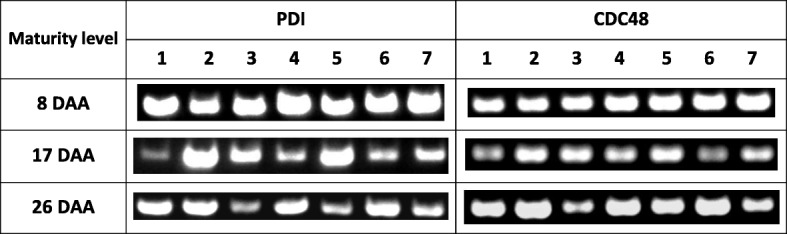
Expression of gene encoding PDI in all collections in relation to expression of gene encoding CDC48 in wheat

(1 - Zyta, 2 - Bamberka, 3 - STH 014, 4 - STH 026, 5 - STH 047, 6 - STH 087, 7 - STH 111)

The products of the PDI gene obtained after electrophoresis of the genotypes belonging to the first collection were defined as very intense. The products obtained for the second collection were generally slightly weaker compared to the first collection. The intensity and size of the products obtained for the samples belonging to the third collection were slightly higher in most cases than for the samples from collection II, but at the same time lower or comparable to collection I. There were slight differences in the expression of the reference gene between cultivars, which was consistent with the differences observed in the experimental gene.

**Fig. 2:**
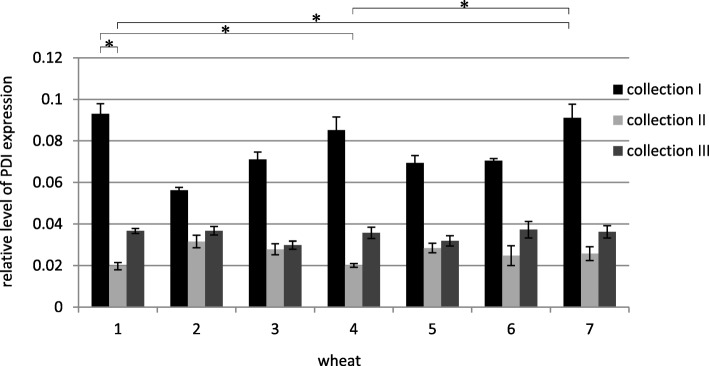
Relative level of PDI expression in all collections – densitometric analysis normalized to the reference gene (logarithmic scale), * *p* < 0.05. (1 - Zyta, 2 - Bamberka, 3 - STH 014, 4 - STH 026, 5 - STH 047, 6 - STH 087, 7 - STH 111). I – 8 DAA, II – 17 DAA, III – 26 DAA

The results of the densitometric analysis for all collections together showed changes in the relative level of expression of the PDI gene during the caryopsis maturation stages discussed in this work. The level of gene expression was the highest in the first collection for each cultivar and line of common wheat. The level of gene expression decreased with caryopsis maturation and slightly increased at the end of the experiment (in collection III) (Fig. 2).

### Quantitative analysis of PDI expression

The thermal-time profile of the reaction and mixture composition were optimized for each gene. Ultimately, the efficiency was in the range of 90–95.3%. Standard curve slope values ranged from − 3.44 to − 3.59. Correlation coefficient values ranged from 0.996 to 1.000.

**Fig. 3:**
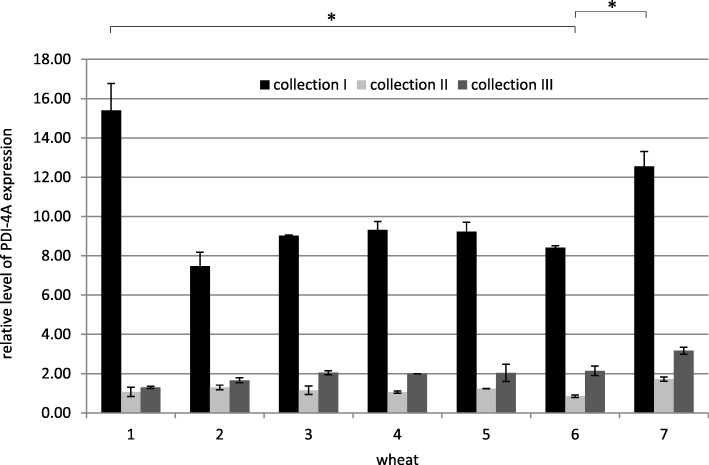
Relative level of PDI-4A expression in all collections. The arithmetic means of all replicates are shown, with the SD marked. * *p* < 0.05. (1 - Zyta, 2 - Bamberka, 3 - STH 014, 4 - STH 026, 5 - STH 047, 6 - STH 087, 7 - STH 111). I – 8 DAA, II – 17 DAA, III – 26 DAA

The comparison of the relative expression level of *PDI-4A* in all examined collections clearly showed that it was the highest in the first collection caryopses at the water ripe stage (Fig. 3). With progression of caryopsis maturation, *PDI-4A* gene expression sharply decreased (milk maturity stage), then it increased again (soft dough stage), however, this increase was insignificant.

**Fig. 4:**
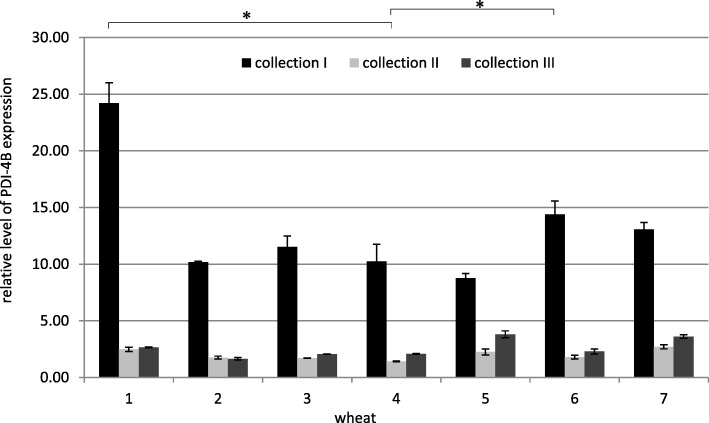
Relative level of PDI-4B expression in all collections. The arithmetic means of all replicates are shown, with the SD marked. * *p* < 0.05. (1 - Zyta, 2 - Bamberka, 3 - STH 014, 4 - STH 026, 5 - STH 047, 6 - STH 087, 7 - STH 111). I – 8 DAA, II – 17 DAA, III – 26 DAA

**Fig. 5:**
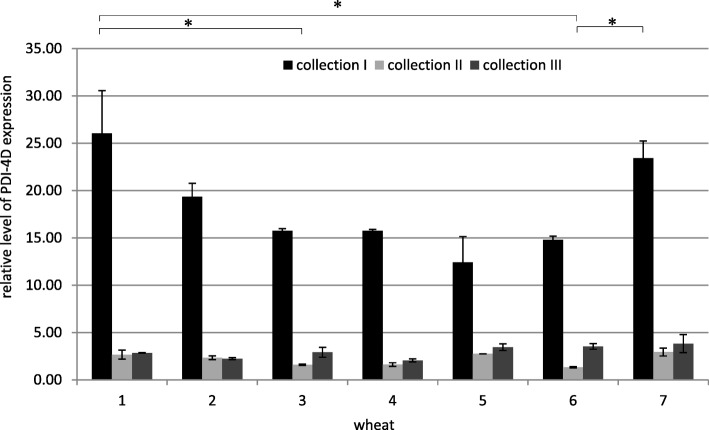
Relative level of PDI-4D expression in all collections. The arithmetic means of all replicates are shown, with the SD marked. * *p* < 0.05. (1 - Zyta, 2 - Bamberka, 3 - STH 014, 4 - STH 026, 5 - STH 047, 6 - STH 087, 7 - STH 111). I – 8 DAA, II – 17 DAA, III – 26 DAA

The comparison of the relative expression level of the *PDI-4B* gene (Fig. 4) in all studied collections allowed to conclusively state that it was the same over time as for the *PDI-4A* gene. The same relationships were observed for the *PDI-4D* gene (Fig. 5).

A statistically significant difference was found for each gene between the relative expression level in the first collection and the level of expression in the second collection and the level of expression between the first and third collection.

**Fig. 6:**
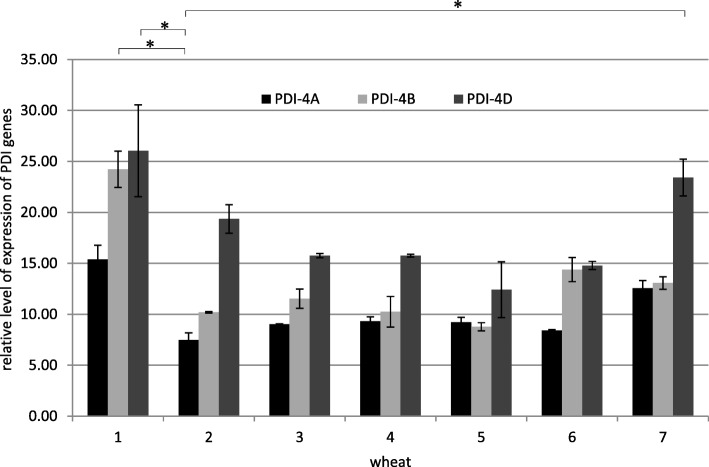
Relative level of expression of *PDI-4A*, *PDI-4B* and *PDI-4D* genes in collection I (8 DAA). The arithmetic means of all replicates are shown, with the SD marked. * *p* < 0.05 (1 - Zyta, 2 - Bamberka, 3 - STH 014, 4 - STH 026, 5 - STH 047, 6 - STH 087, 7 - STH 111)

Relationships observed between individual genes’ expression in each of the collections were similar, thus the following list was presented for the first collection (Fig. 6). Despite different expression levels of each gene between the tested wheat genotypes, it could be noticed that the level of *PDI-4D* gene expression was higher in the first collection compared to the level of expression of remaining genes, and this applied to all tested wheats. The lowest level of expression for the majority of the genotypes tested was noticed for the *PDI-4A* gene. The cultivar Zyta was primarily distinguished by high level of expression of all genes, and was followed by the STH 111 line. None of the tested wheat genotypes was characterized by clearly lower expression levels of all genes simultaneously, compared to other genotypes.

The most statistically significant differences were observed in the first collection compared to the others. A highly statistically significant difference was found between the relative expression levels of *PDI-4A* and *PDI-4D*, and *PDI-4B* and *PDI-4D* genes. A statistically significant difference was demonstrated between the expression level of *PDI-4A* and *PDI-4B*.

## Discussion

Electrophoregrams obtained in a semi-quantitative analysis showing the amplification products for the genotypes belonging to the first collection (8 DAA) indicated a high expression level of protein disulfide isomerase (Table 1). Similar conclusions applied to quantitative analysis. The results obtained for the genotypes in the second collection indicated a lower relative level of expression of the three homeologous genes examined. The results obtained for the third collection indicated in most cases a slightly higher level of PDI expression than in the second collection. The highest level of PDI expression was recorded at the early stage of caryopsis maturity (water ripe stage). As caryopsis matured, it entered the stage of milk maturity, the PDI expression was then quite significantly reduced and only slightly increased at the soft dough stage. This dependence applied to all tested wheat cultivars and lines. Literature data indicate that a high level of PDI expression is fairly quickly reached in caryopsis development, preceding the beginning of the process of storage protein accumulation by several days [22, 23]. Shimoni et al. [22] observed the beginning of gliadin accumulation in the wheat endosperm at ca. 15–17 DAA using Western blotting. According to them, the level of PDI expression increased significantly between 5 and 9 DAA. Grimwade et al. [23] showed the presence of gluten protein transcripts at 11 DAA and protein expression from 14 DAA. SDS-PAGE electrophoresis performed by Du Pont et al. [24] allowed to show the presence of gluten proteins already at 12 DAA, while the Western blot technique allowed to demonstrate the high level of expression of PDI protein already at 5 DAA, which persisted up to approx. 12 DAA, and at 18 DAA markedly declined. At the transcriptome level, the highest level of PDI expression was found between 6 and 10 DAA. D’Aloisio et al. [25] found a quite high level of PDI expression from 4 to 16 DAA on the basis of Northern blot analyses. The highest transcript level was recorded on 8–12 DAA. The PDI expression was clearly lower during 20–24 DAA. Quantitative analysis (real-time PCR) carried out by D’Aloisio et al. [26] showed, similarly to the current work, that the level of PDI expression was the highest at the early stage of caryopsis development (10 DAA) (Figs. 3, 4 and 5). Then, the level of expression quite rapidly decreased between 15 and 21 DAA, which coincided with the beginning of the seed filling phase – starch and gluten protein deposition. Singh et al. [27] conducted a wheat transcriptome analysis using microarrays to identify genes whose expression level changes at three stages of grain development (7, 14 and 28 DAA) and depending on the cultivar quality. These authors focused on the sequences whose expression level changed at least 10-fold. Most of the sequences identified in BLAST, whose expression changed significantly during grain development between qualitatively good and inferior cultivars, were responsible for coding of the storage gluten proteins. The coding sequences of gliadins and glutenins exhibited different levels of expression between qualitatively good and inferior cultivars (in favor of the former) at an early stage of grain development (7 DAA). Such large differences were not observed in the later stages of grain development (14, 28 DAA), which confirmed the importance of the early stages of grain maturation in obtaining good baking quality. Laudencia-Chingcuanco et al. [28] found that a significant number of genes exhibited changes in the level of expression during grain development and these changes were most significant at early developmental stages, i.e., between 7 and 14 DAA, which coincided with the beginning of the grain filling process. It can be confirmed that the level of PDI expression in tested wheat cultivars and lines was the highest during the grain filling phase (usually at its early stage). The level of PDI gene expression was the highest at 8 DAA, thus, as expected, intense PDI expression preceded the moment of the most intense accumulation of storage substances.

There were no significant differences in the intensity (concentration) of products between electrophoregrams obtained in a semi-quantitative analysis showing the amplification products for genotypes belonging to the first collection, while some diversification between genotypes was demonstrated by densitometric analysis (Fig. 2). Genotypes that were distinguished by the level of PDI expression in the first collection did not differ in subsequent collections.

The PDI expression was evaluated between individual wheat genotypes on the basis of data gathered from all collections considering that the tested wheat cultivars and lines were characterized by different genotype-phenotype combinations (i.e., HMW-GS set and the qualitative classification). The characteristic genotype, indicating the set of HMW glutenin subunits carried by particular wheat was taken into account. This was done in order to establish a potential correlation between the level of PDI expression and the flour baking value, expressed in classifying a given cultivar or line into a qualitative group. Unfortunately, the analysis did not allow to clearly indicate wheat cultivar or line, which would be characterized by the lowest expression level of protein disulfide isomerase. Similarly, it was difficult to pinpoint wheat with the highest level of PDI expression while analyzing electrophoregrams and densitometric measurements.

The cultivar Zyta and the STH 111 line, as Bamberka and STH 047, had 7 points on the Payne scale and the same HMW glutenin subunits. As in the case of Bamberka (7 points, A/E), the high level of PDI expression in the semi-quantitative analysis was noticed only in two collections, thus the potential correlation of PDI expression with baking quality of flour was not clear. Moreover, PDI expression for the STH 111 line remained at a high or an average level, and this line was included only in the quality class C, i.e., intended for feed. Similarly, the level of PDI expression for the STH 026 and STH 087 lines was not stable in the subsequent stages of the experiment. After comparing PDI expression in genotypes with the same HMW-GS combination, such a comparison was performed for wheat genotypes representing different genotype-phenotype combinations (HMW-GS set and classification to qualitative group). There was no close correlation of the PDI expression level with the wheat utility value based on the obtained data.

It was difficult to draw valid conclusions based on the results of the semi-quantitative analysis. However, since some differences in the level of expression were observed, the research was continued using quantitative analysis – real-time PCR with primers specific to homeologous sequences. The most important issue, when designing their sequences, was to include as many single nucleotide polymorphisms (SNPs) as possible in order to exclude the amplification of sequences from a different genome than the one under study. This approach also resulted in very frequent genotypes that were distinguished by the level of PDI expression in the first collection, but they did not differ in subsequent collections. The current study failed to identify a genotype characterized by the unmistakably lowest level of expression of PDI homeologous genes. However, Zyta and STH 111 were characterized by a higher level of expression of the examined genes in the vast majority of collections in comparison to other cultivars and lines (Figs. 3, 5).

The STH 111 line showed a high level of *PDI-4A* gene expression throughout the experiment compared to other wheat genotypes, although it belonged to the qualitative group C (feed) (Fig. 3). Zyta of the same genotype, but classified into groups A/E, was characterized in subsequent collections by a high, average and low level of *PDI-4A* gene expression, respectively. The *PDI-4A* expression level for the “unfavorable genotype + unfavorable phenotype” combination, may be average (STH 047) or high (STH 111) at similar utility value (B/C). The average level of *PDI-4A* expression for the mixed case “favorable genotype + unfavorable phenotype”, (STH 014) seemed to be important for wheat quality, but also the average expression level of this gene was noted in the mentioned STH 026 and STH 087 lines with a good genotype and highly classified. Thus, *PDI-4A* expression level did not correlate with the utility value of wheat and its high level was not a guarantor of a high-quality cultivar/line. The presence of unfavorable, but also valuable subunits, the low or average level of *PDI-4A* expression does not have to be an obstacle to the quality of a given cultivar or line.

*PDI-4B* gene expression in Bamberka was characterized by an intermediate or slightly lower level compared to others, which did not interfere with the high quality of this cultivar (A/E) (Fig. 4). The situation was reversed for the STH 047 line, which was classified lower (B/C) and the *PDI-4B* expression was the lowest in the first collection among other wheats, while in the other collections it was high. A generally average *PDI-4B* transcription level was found for STH 026 and STH 087 lines (favorable genotype and phenotype) in comparison to other wheats, which in the presence of average or qualitatively unfavorable subunits in the genotype did not prevent high classification of these lines. Although the level of *PDI-4B* expression was average or high in the case of STH 047 and STH 111 lines (unfavorable genotype and phenotype), both lines could be intended for feed. The level of *PDI-4B* expression was intermediate for the mixed “unfavorable genotype + favorable phenotype” Bamberka variant, and yet it was a high quality cultivar. Zyta in turn was characterized by high level of *PDI-4B* expression and good quality. Nevertheless, the discrepancy between the results obtained within the same experimental variant did not allow drawing valid conclusions regarding the beneficial indirect effect of *PDI-4B* expression on wheat baking value. The *PDI-4B* expression also did not explain the discrepancies between the valuable baking genotype and the low value of the flour obtained in practice.

STH 111 and Zyta were characterized by the high level of *PDI-4D* expression throughout the experiment compared to other wheats, nevertheless they were classified to the opposite qualitative groups, which rather excluded the dominant role of *PDI-4D* in the final shaping of the baking value (Fig. 5). Bamberka and STH 047 had an ambiguous, but similar expression profile of the *PDI-4D* gene, but a different classification to the qualitative group. An average or low level of *PDI-4D* expression was found for STH 026 and STH 087 lines (favorable genotype and phenotype) in comparison to other cultivars and lines, which in the presence of preferred subunits in genome D did not negatively affect the utility value (A/E). The level of *PDI-4D* gene expression was generally high for the “unfavorable genotype + unfavorable phenotype” combination (STH 047 and STH 111). The level of *PDI-4D* gene expression was generally intermediate for the mixed case (favorable genotype + unfavorable phenotype; line STH 014) compared to other wheat genotypes. This may be important in the context of wheat quality, because this wheat has a total of 10 Payne quality points, and the Dx5 + Dy10 set in genome D, but is classified low in terms of utility (C/K). Nevertheless, *PDI-4D* expression level was comparable to that observed in STH 026 and STH 087 with a good genotype and highly classified. The level of expression of this gene, similarly as the previously discussed homeologous genes, was not the most important factor determining the baking value in the event of discrepancies between the baking genotype and flour value obtained in practice.

The *PDI-4D* gene had the highest level of expression in all collections for all (or most) wheats, followed by *PDI-4B* and finally *PDI-4A* (Fig. 6). The results indicated the dominant expression of the disulfide isomerase gene belonging to genome D, as compared to other homeologous genes of genomes A and B. While examples of PDI expression studies that do not include differences resulting from homeologous PDI sequence variants are common in the literature, works that consider these polymorphisms are scarce. Ciaffi et al. [16] studied the expression of PDI homeologous genes. They conducted a semi-quantitative analysis using caryopses at various stages of maturity. The expression profile of all PDI homeologous genes changed over time in the same manner. The researchers pointed to the lack of differences in the expression of these genes, which was not consistent with the results of this work of both semi-quantitative and quantitative analysis. Nevertheless, they applied a different, less sensitive research technique.

The literature on PDI is mainly focused on the evaluation of gene expression level – or significantly less frequently – the expression of homeologous genes encoding it. The genotype is also examined for the presence of good and inferior baking value markers, and sometimes such analyses are combined with technological studies. However, there are virtually no examples of combining these types of experiments together with the analysis of disulfide isomerase expression. Only Dong and co-authors attempted such experiments [29], and analyzed common wheat lines for the expression of PDI encoding gene and several genes encoding proteins from the PDI-like family. A better gluten content or bread volume and more favorable parameter values were found for the line containing the Dx5 + Dy10 set during rheological tests. The results presented by scientists depicting the expression of the PDI-encoding gene and PDI-like proteins showed that its level changed over time and these changes did not always occur simultaneously between the forms with almost the same baking genotypes. Moreover, the dynamics of changes in individual forms and fluctuations in the level of expression may also differ (e.g., the level of expression increases in one line, while in another it remains the same).

The changes occurring in the transcriptome and proteome of developing caryopses were often the subject of research. Laudencia-Chingcuanco et al. [28] found statistically significant differences in the level of expression of more than 2.000 genes at six stages of caryopsis development, between 3 and 35 DAA. No genes encoding PDI or genes of this family were identified in this group, while the coding sequence of the thioredoxin gene with maximum expression at 7 DAA was identified. Guo et al. [30], using two-dimensional electrophoresis and mass spectrometry techniques, focused on the proteome of two cultivars with different gluten quality, whose grains were collectioned at five stages of maturity during 6–31 DAA. Researchers analyzed what proteins appeared in both cultivars at successive stages and which differences in cultivar expression were statistically significant. Similarly, no protein of the PDI family was indicated in the latter study. Interesting results were also provided by Singh et al. [27], who performed the analysis of wheat transcriptome using microarray technology. The seeds were collectioned at three time intervals (7, 14, 28 DAA) from four cultivars – two with favorable baking characteristics and two with unfavorable traits. The aim of that study was to identify genes whose expression varies statistically significantly in three experimental variants: depending on the quality of the cultivar, the stage of grain development and both of these factors together. The focus was on sequences whose expression level was at least 10-fold lower or higher between qualitatively good and inferior cultivars in at least one experimental variant. However, PDI proteins were not specified in any experimental variants, which indicated that PDI expression was not such an important factor for the quality of individual cultivars.

## Conclusions

The current study attempted to resolve the potential impact of PDI gene expression on the flour baking value due to the significant role of PDI in protein maturation. Nevertheless, the results obtained did not allow to conclusively state such a strong dependence. It is worth to perform such research in the future on a larger research pool in order to confirm the results obtained in this study. Analyzing the results of this work in relation to the available literature, we concluded that the reasons for discrepancies between the expected HMW glutenin genotype and the utility value of wheat obtained in practice should, therefore, be sought not on the disulfide isomerase side, but other proteome components of individual wheat genotypes. Low-molecular subunits (LMW-GS), which are an important structural fraction of the polymeric gluten network and form its scaffold, constitute almost one-third of gluten proteins. Literature data indicate a great diversity of this fraction. There are examples of studies in the available literature on the effect of particular LMW alleles and values of certain technological parameters [6, 31]. The potential impact of the LMW-GS fraction is pointed out in the instances of discrepancies between the technological and molecular research results [32].

## Methods

### Plant material

The experimental material was obtained from the Stacja Hodowli Roślin Strzelce Sp. z o.o. The IHAR group. The Station added a description for each wheat cultivar and line containing the composition of HMW-GS divided into genomes (A/B/D) and baking quality, which was marked as a specific group in terms of technological value and intended use (qualitative groups according to COBORU: A, B, C, E and K) (Table 2).

**Table 2:** Characterization of the material used for the experiment

Wheat cultivar/line	HMW-GS composition (A/B/D)	Quality score (A/B/D)	Quality group (COBORU)
Zyta	2*/7 + 9/2 + 12	3/2/2 = 7	A/E
Bamberka	N/7 + 9/5 + 10	1/2/4 = 7	A/E
STH 014	2*/7 + 8/5 + 10	3/3/4 = 10	C/K
STH 026	1/7 + 9/5 + 10	3/2/4 = 9	A/E
STH 047	N/7 + 9/5 + 10	1/2/4 = 7	B/C
STH 087	1/7 + 9; 6 + 8/5 + 10	3/2; 1/4 = 9;8	A/E
STH 111	2*/7 + 9/2 + 12	3/2/2 = 7	C

The experimental materials were the seeds of two cultivars and five lines of winter wheat (cultivars Zyta and Bamberka and lines: STH 014, STH 026, STH 047, STH 087 and STH 111), because the highest protein expression of the disulfide isomerase gene (in three wheat genomes) was observed in developing caryopses, as compared to various organs, and storage gluten proteins are located in the endosperm. As regards the cultivation, it is important to obtain wheat cultivars with a high baking quality and nutritional value, therefore, it is necessary to type the lines with desirable utility traits, as part of breeding selection and work on new cultivars. Hence, we also included several wheat lines in this study. The cultivars and lines of common wheat were specifically selected for the purpose of this work. They represent different experimental variants, i.e., they are characterized by different genotype-phenotype combinations. The study analyzed wheat classified as producing a good quality flour, whose baking genotype has a high total number of points on the qualitative scale (STH 026 and STH 087), as well as wheat giving a worse quality flour, whose baking genotype has a lower total number of points in the qualitative scale (STH 047 and STH 111). ‘Mixed’ cases were also analyzed, i.e., good baking genotypes, but classified as those with a worse quality flour (STH 014) and cultivars Zyta and Bamberka, which, despite the relatively low total number of points in the qualitative scale for the genotype, were classified as valuable in terms of flour quality.

Some wheats within the same experimental variant differed in genotype. Although they had the same total number of quality points, their genotype was conditioned by other HMW-GS subunits. It should also be noted that wheats with exactly the same baking genotype are sometimes classified by the Station into different quality groups.

The seeds of all wheat genotypes were sown in pots in the Vegetation Hall. The seeds of each cultivar and line were collected (by cutting ears) at three different time intervals, counting the number of days after anthesis (DAA), because PDI gene expression does not remain at the same level during grain formation and maturation. Three research pools were created this way, and they were referred to as collections. The first collection took place 8 days after anthesis (8 DAA, collection I). Subsequently the grain was collected on 17 DAA (collection II) and 26 DAA (collection III). A pool of ears was dedicated for a given collection for each cultivar and line. The gradually emerging ears were properly marked and the anthesis progress was recorded. After the predetermined time, individual ears were cut, wrapped, labeled and immediately placed in a dry ice dewar before storing at − 70 °C until nucleic acid isolation. Immature caryopses obtained from main ears were used in the experiments. Caryopses from the first collection were obtained in the watery ripe stage (BBCH 71). Collection II was represented by caryopses of the late milk maturity stage (BBCH 77). Caryopses from the last collection entered the soft dough stage (BBCH 85). For comparison, ready-to-collection caryopses are in the harvest ripe stage (BBCH 92).

### Semi-quantitative analysis of PDI expression

RNA isolation was performed in triplicate for each wheat cultivar/line from each collection, using the GeneMATRIX Universal RNA Purification Kit (EURx). The isolated RNA was stored at − 80 °C. DNase I (Novazym) was used in order to effectively eliminate DNA during RNA isolation. Spectrophotometric measurements were made using the NanoDrop 2000c system (Thermo Scientific) to assess RNA purity. Electrophoresis was performed under denaturing conditions in a 1.5% denaturizing agarose gel for one hour at 90 V to assess the quality of the isolated RNA.

Two-stage RT-PCR was performed using the Revert Aid First Strand cDNA Synthesis Kit (Thermo Scientific) and oligo (dT) primers and an RNase inhibitor in order to conduct semi-quantitative analysis of PDI expression. The reaction was carried out in three replicates for each collection (biological and technical), according to the manufacturer’s protocol. The obtained cDNA was a template for the PCR reaction. Primers were designed in Primer-BLAST (Basic Local Alignment Search Tool), using gene coding sequence deposited in the GenBank database (accession number HQ911363.1). The primer sequences flanked exon-exon junctions. These were forward: 5’-TTGGCACCTGAGTATGAGAAG-3′, and reverse: 5’-TTCAATAAGAACATTTTTGCCAGAT-3′ primers, respectively. The expected length of the product was 999 bp. The reaction mixture (25 μl) contained cDNA (1 μl) and the following components in final concentrations: primers (0.2 μM), dNTP mix (0.2 μM), reaction buffer (1x), DreamTaq polymerase (Thermo Scientific) (1 U). Amplification reactions were run in triplicate in a C1000 Touch Thermal Cycler (Bio-Rad) in a thermal-time profile by Johnson and Bhave (2004) with annealing temperature of 52 °C.

A gene coding for one of the cell cycle control proteins, CDC48 (referred to in the publications with the Ta54227 UniGene accession number, in the GenBank database – EU267938) was used as the reference gene for the semi-quantitative analysis, because it was characterized by a stable expression [33–35]. The primers designed by Paolacci et al. [33] were used for its amplification, and they generated amplicons of 227 bp in size. The composition of the reaction mixture for the amplification of CDC48 gene fragments and the thermal-time profile were the same as for the experimental gene (taking into account the primer annealing temperature established for the gene – 63 °C). The amplification products were separated in a 1.5% agarose gel (Prona agarose). Electrophoresis was performed in 1 × TBE buffer (0.89 M Tris, 0.89 M boric acid, 2 mM EDTA, Sigma-Aldrich) for 1.5–2 h at 90 V. The analysis of product sizes was carried out using the Quantity One® program (Bio-Rad).

The relative number of transcripts of a given gene was assessed using densitometric analysis in the Quantity One® program (Bio-Rad). The results obtained for the tested gene were normalized to the data for the reference gene. Calculations were performed using the arithmetic mean of three biological replicates, taking into account the standard deviation.

### Quantitative analysis of PDI expression

Luminaris HiGreen qPCR Master Mix (Thermo Scientific) was used for quantitative analysis of gene expression. SYBR Green I dye was present in the mixture as well as a buffer which, when added in the appropriate volume, provided the final MgCl_2_ concentration of 2.5 mM. Hot Start Taq DNA polymerase was used. The cDNA obtained by reverse transcription (see subsection 5.2.) served as a template for quantitative analysis. Allelospecific primers were used for real-time PCR amplification of *PDI-4A*, *PDI-4B* and *PDI-4D* gene fragments. Primers were designed using the Primer-BLAST tool and the cDNA sequence for three PDI genes deposited in GenBank (accession numbers: AF262979.1, AF262980.1 and AF262981.1). The MultAlin program was used to compare sequences. The applicable rules for real-time PCR were followed when designing primers. Primers, due to the use of cDNA as a template, flanked the exon-exon junctions, to exclude the possibility of accidental DNA amplification. The characteristics of primers for PDI genes are given in Table 3.

**Table 3:** Characterization of primers used for quantitative analysis for three PDI genes in wheat

Gene	Name	Sequence 5′-3′	Product size	Annealing temp.
*PDI-4A*	QAF	CATCCTTACCTCTTGAAATACTTT	194 bp	57 °C
	QAR	TTCAGGCCAAAGTACTGGAAG		
*PDI-4B*	QBF	CGGCAAGGATGTCAAGTTCCTA	200 bp	58 °C
	QBR	TGAATGGTGTCAATTTGCCATCG		
*PDI-4D*	QDF	CCTCCTGAAATTCTTCCAGAC	186 bp	56 °C
	QDR	TTCAGCCCGAAGTACTGGAAA		

The mfold program was used to generate possible structural models based on a provided sequence to test the probability of secondary structure occurrence in primers and amplicons [36]. In addition, the possibility of primer-dimer formation was tested using the Multiple Primer Analyzer (Thermo Scientific) and Oligo Analyzer (Integrated DNA Technologies). When designing primers, the possibility of amplification sequences other than target sequences was also excluded by verification with blastn (Nucleotide BLAST; Basic Local Alignment Search Tool). The reliability of the primers (traditional PCR, real-time PCR, sequencing) was checked prior to the actual analysis. Standard curves were prepared using two-fold dilutions of the pool of all cDNAs to determine the reaction yield. Amplification occurred most effectively at the 0.3 μM final primer concentration for the *PDI-4A* gene, 0.4 μM for the *PDI-4B* gene and 0.4 μM for the *PDI-4D* gene, and these concentrations were finally used in the reaction mixtures. All amplification reactions were run in triplicate (biological and technical replications) in CFX96 TouchTM Real-Time PCR Detection System thermocycler (Bio-Rad) in 96-well plates. To the reaction mixture (10 μl), 0.6 μl cDNA and reaction buffer (1x) were added. The thermal-time profile was used according to the manufacturer’s recommendation, taking into account the annealing temperature for individual genes and supplementing it by adding a step after each reaction allowing calculating the melting curve: 95 °C for 10 s and melting analysis (increase from 65 to 95 °C – 0.5 °C/5 s). No RT control and no template control, NTC, were also included.

Two reference genes were selected for the quantitative analysis, and the results obtained for them were averaged [37]. The first one was the CDC48 gene, which was previously used for semi-quantitative analysis, together with the primers used previously, whereas the second – the gene coding for actin – whose cDNA sequence is in the GenBank database (accession number: AB181991.1), and which was used for wheat quantitative analyses [38, 39]. The primers were designed using the Primer-BLAST tool: forward primer: 5’-CCCAGCAATGTATGTCGCAA-3′, reverse primer: 5’-GAGGAAGCGTGTATCCCTCA-3′, with an expected product size of 131 bp and annealing temperature of 55 °C. A similar procedure of primer verification was used for the reference genes as for the experimental genes. Amplification occurred most effectively at primer concentrations of 0.5 μM for the actin gene, 0.8 μM for the CDC48 gene and these concentrations were finally used in the reaction mixtures. The conditions and profile of the reactions were the same as for the experimental genes, taking into account the respective concentrations of the primers and the specific annealing temperature for a given gene.

The purified nested PCR products were used as standards to assess the level of gene expression. First stage primers for the experimental and reference genes were designed using the Primer-BLAST tool based on the sequences used to design real-time PCR primers. Binding sites of the external primers for the experimental genes (*PDI-4A*, *PDI-4B* and *PDI-4D*) were designed on the basis of identical fragments for these three genes (excluding SNP sites). Their characteristics are presented in Table 4. They were also validated for specificity.

**Table 4:** Characterization of outer primers used for nested PCR in wheat

Gene	Name	Sequence 5′-3′	Product size	Annealing temp.
*PDI*	QFnest	GAGTATGAGAAGGCGGCCC	983 bp	51 °C
	QRnest	AGAACATTTTTGCCAGATTTGA		
*actin*	AFnest	ACAACTGGGATGACATGGGGAA	435 bp	53 °C
	ARnest	CAAGGGCCACGTAAGCGA		
*CDC48*	CFnest	TTAGGCCAGGGCGTCTTGA	542 bp	54 °C
	CRnest	AGTGAAGGGCACAACAACGAG		

The amplification products purified with the GeneMATRIX PCR/DNA Clean-Up Purification Kit (EURx) were used as standards, whose subsequent ten-fold dilutions were real-time PCR templates to generate standard curves. Dilution standards were amplified on plates simultaneously with the experimental samples analyzed for a given gene. Spectrophotometric measurements were carried out and the number of copies of individual genes was calculated [40–42].

Two reference genes were used in this work, and the relative expression level of the experimental genes was presented in diagrams representing the arithmetic mean of the replicates, taking into account the standard deviation (SD). Besides the analysis of homeologous gene expression level at different stages of caryopsis maturity, additionally a comparison of the expression level between these genes in subsequent collections was performed.

### Statistical analysis

Statistical analysis of semi-quantitative and quantitative analysis results was carried out using Statistica ver. 12 (StatSoft). The normality of variable distributions was verified using the Shapiro-Wilk test. The Levene test was performed to verify variance homogeneity. The data were analyzed using non-parametric tests when they did not show normal distribution and when the variance was not homogeneous. The observed differences were analyzed using the Kruskal-Wallis test with a multiple comparison test. Statistically significant differences were assumed at the significance level of *p* ≤ 0.05, whereas the differences were highly statistically significant at *p* ≤ 0.01. The results obtained for individual wheat genotypes were compiled together in subsequent collections in the quantitative analysis to compare the expression level of the homeologous genes at each stage of caryopsis development. Additional statistical analyses were carried out for each gene, taking into account data from individual collections without distinguishing specific wheat genotypes. Lastly, additional statistical analyses were conducted within each collection that included data for genes without distinguishing specific wheat genotypes to compare the expression of individual genes with each other over time.
